# Guidance of interventions in structural heart disease; three-dimensional techniques are here to stay

**DOI:** 10.1007/s12471-016-0945-0

**Published:** 2017-01-17

**Authors:** M. Voskuil, H. Sievert, F. Arslan

**Affiliations:** 1 Department of Cardiology, University Medical Center Utrecht, Utrecht, The Netherlands; 2 CardioVascular Center Frankfurt, Frankfurt, Germany; 3 Laboratory of Experimental Cardiology, University Medical Center Utrecht, Utrecht, The Netherlands

A significant part of current daily practice in interventional cardiology consists of interventions in structural heart disease. This involves a diversity of interventions in both acquired and congenital heart defects. A safe and predictable intervention can be a challenge, particularly in patients with congenital heart disease who often have multiple operations in their medical history. Improvements in device technology have shown enhanced procedural and clinical outcome in this often fragile patient group. In addition to technological progress, new imaging tools have emerged for the improvement of patient selection, procedural planning and guidance. Three-dimensional (3D) imaging techniques using echocardiography, computed tomography (CT) or magnetic resonance (MR) for visualisation of the cardiovascular system were introduced in the ’80s and ’90s [1, 2]. In the late ’90s 3D rotational angiography (3DRA) was developed, but was mainly used in neuroradiology procedures [3]. It was only after 2000 that the first manuscripts concerning the use of 3D angiography for coronary anatomy and congenital heart disease appeared [4, 5]. Remarkably, in the first description of 3DRA, the patient was rotated around the radiation source instead of the other way around [6]. An increasing number of centres are currently incorporating 3D techniques using MR/CT, (transesophageal) echocardiography and/or rotational angiography in their daily clinical practice. Moreover, 3D printing has emerged as an additional tool for this patient cohort. Using 3D printing, patient-specific implants and devices can be designed and tested, opening new horizons in personalised patient care and cardiovascular research. Furthermore, physicians can better elucidate anatomical abnormalities with the use of 3D-printed models and improve communication with their patients.

In this special issue of the *Netherlands Heart Journal* on imaging and interventions in structural heart disease, it is striking that a substantial number of the submitted manuscripts concern these 3D imaging and printing techniques [7–10]. This emphasises the keen interest of researchers and clinicians in the current developments in imaging for structural heart disease. *Goreczny et al.* describe the additional value of 3D in the guidance of percutaneous pulmonary valve implantation (PPVI) that resulted in a reduction in exposure to contrast and radiation when compared with traditional 2D guidance [7]. These findings are in line with a recent paper from *Starmans et al.*, who showed the diagnostic quality of 3DRA to be superior in children with an aortic coarctation, with less radiation exposure than conventional angiography (CA) [11]. Furthermore, in the current issue *Pockett et al.* state that during PPVI, 3DRA may facilitate higher procedural success and decrease the risk of serious adverse events such as coronary artery compression [8]. As in many studies on congenital heart disease, it is difficult to test the additional value of 3DRA compared with CA in a true randomised or controlled study. Nevertheless, the studies mentioned earlier seem to confirm the experience of the users of 3DRA, i. e. that this technique enhances procedural safety and technical outcome. In mainstream coronary artery intervention CA techniques still seem adequate for an efficacious and safe outcome of the procedure. However, the limitations of CA include the simultaneous opacification of overlying structures, foreshortening of structures, and the inability to visualise structures without injection of contrast. Integration of 3D image data sets with fluoroscopy seems to overcome the majority of these limitations. Therefore, 3DRA appears to have become indispensable in interventions in structural heart disease. One major limitation of the use of 3D imaging techniques for fluoroscopic roadmaps during an intervention is that these images are not acquired in real time [12]. Therefore, any change in patient position or distortion of the anatomy by ridged interventional equipment (wires, balloons etc.) can cause misalignment of the images. However, correction of this potential misalignment after placement of the equipment in the catheterisation laboratory is possible and therefore mandatory. One of the latest developments involves printed models to replicate complex cardiovascular structures and their surroundings in patients with structural and congenital heart disease. *Meier et al.* discuss the different steps of the 3D printing process, which include image acquisition, segmentation, printing methods and materials used [9]. Alternatively, computer 3D simulation using CT images has the ability to predict frame geometry, aortic leaflet calcium displacement and paravalvular leakage after transcatheter aortic valve implantation, as shown by *El Faquir et al*. [10]. Thus, comparable with the use of 3D printed models, computer simulation with CT can guide the operator in the choice of type and size of the valve.

In addition to 3D technology, the fusion of different imaging techniques shows potential for the planning and guidance of interventions. This is also represented in the manuscript of *Afzal et al.*, who describe the real-time fusion of fluoroscopic and echocardiographic images using the EchoNavigator system [13]. They used this technique to optimise the safety and efficacy of their transseptal puncture for MitraClip implantation and left atrial appendage closure. In these procedures, the location of the puncture site in a prespecified region of the interatrial septum is essential for the length and final outcome of the procedure. Indeed, they showed a decrease of procedural time using this system in comparison with the conventional technique, most likely probably only guided by fluoroscopy.

In conclusion, 3D imaging techniques in combination with the integration of imaging modalities provide additional information which can be used for device choice and procedure guidance in patients with structural heart disease. As also reflected by the manuscripts in this issue of the *Netherlands Heart Journal*, these interventions can be performed safely and with a good technical outcome, without exposing patients to higher doses of radiation. It is likely that radiation usage in 3DRA will be further reduced, favouring the use of this technique in daily clinical practice.

## References

[1] Nixon JV, Saffer SI, Lipscomb K, Blomqvist CG (1983). Three-dimensional echoventriculography. Am Heart J.

[2] Hale JD, Valk PE, Watts JC (1985). MR imaging of blood vessels using three-dimensional reconstruction: methodology. Radiology.

[3] Anxionnat R, Bracard S, Macho J (1998). 3D angiography. Clinical interest. First applications in interventional neuroradiology. J Neuroradiol.

[4] Raman SV, Morford R, Neff M (2004). Rotational X‑ray coronary angiography. Catheter Cardiovasc Interv.

[5] Boccalandro F, De La Guardia B (2001). Smalling RW. Rotational Aortogram With Three-Dimensional Reconstruction in a Case of Repaired Aortic Coarctation. Circulation.

[6] Schad N, Brunner H (1966). Cineangiography of congenital heart defects with rotation of the patient]. Med Audio Vis. German.

[7] Moszura T, Dryzek P, Lukaszweski M, Krawczuk A, Moll J, Morgan GJ (2017). Three-dimensional image fusion guidance of percutaneous pulmonary valve implantation to reduce radiation exposure and contrast dose. Neth Heart J.

[8] Pockett C, Moore JW (2017). Three dimensional Rotational Angiography For Assessment Of Coronary Arteries During Melody Valve Implantation: Introducing A Technique That May Improve Outcomes. Neth Heart J.

[9] Meier LM, Meineri M, Qua Hiansen J, Horlick EM. Structural and Congenital Heart Disease Interventions: The Role of 3D Printing. Neth Heart J. 2017;25. Doi:10.1007/s12471-016-0942-3. 10.1007/s12471-016-0942-3 PMC5260628 28083857

[10] El Faquir N, Ren B, van Mieghem NM, Bosmans J (2017). Patient-specific Computer Modeling – Its role in the Planning of Transcatheter Aortic Valve Implantation. Neth Heart J.

[11] Starmans NL, Krings GJ, Molenschot MM, van der Stelt F, Breur JM (2016). Three-dimensional rotational angiography in children with an aortic coarctation. Neth Heart J.

[12] Fagan TE, Truong UT, Jone PN (2014). Multimodality 3‑dimensional image integration for congenital cardiac catheterization. Methodist Debakey Cardiovasc J.

[13] Afzal S, Veulemans V, Balzer J, et al. Safety and efficacy of Transseptal Puncture guided by Real-Time Fusion of Echocardiography and Fluoroscopy. Neth Heart J. 2017;25. Doi:10.1007/s12471-016-0937-0. 10.1007/s12471-016-0937-0 PMC5260626 27966185

